# Timing of Microaxial Flow Pump Support and In-Hospital Mortality in Infarct-Related Cardiogenic Shock: The TIMING-CS Study

**DOI:** 10.3390/jcdd13100498

**Published:** 2026-10-03

**Authors:** Klevis Mihali, Julian Kreutz, Birgit Markus, Yannick Massmann, Lukas Harbaum, Georgios Chatzis, Maryana Choukeir, Styliani Syntila, Bernhard Schieffer, Philipp Lauten

**Affiliations:** 1 Department of Cardiology, Angiology, and Intensive Care Medicine, Philipps-Universität Marburg, Baldingerstrasse, 35043 Marburg, Germanybirgit.markus@staff.uni-marburg.de (B.M.); philipp.lauten@me.com (P.L.); 2 Department of Cardiology, Heart Center, Central Hospital of Bad Berka, 99437 Bad Berka, Germany

**Keywords:** cardiogenic shock, acute myocardial infarction, microaxial flow pump, temporary mechanical circulatory support, landmark analysis

## Abstract

(1) Background: The association between microaxial flow pump (mAFP) timing and outcomes in acute myocardial infarction-related cardiogenic shock (AMI-CS) remains uncertain. (2) Methods: We conducted a retrospective single-center study of patients with AMI-CS admitted between January 2017 and July 2025. Prespecified 24 h landmark analyses included patients alive 24 h after intensive care unit (ICU) admission. The primary analysis compared patients with ST-segment elevation myocardial infarction (STEMI) receiving mAFP within 6 h with STEMI patients managed without temporary mechanical circulatory support (tMCS). Later STEMI implantation (>6–≤24 h) and both non-ST-segment elevation myocardial infarction (NSTEMI) timing cohorts were exploratory. The primary endpoint was subsequent in-hospital mortality from the landmark onward. Associations were assessed using multivariable logistic regression and propensity score-weighted models. (3) Results: In the primary STEMI cohort, 106 patients received mAFP within 6 h and 59 received no-tMCS. Subsequeunt in-hospital mortality was 17.0% versus 23.7%, respectively (*p* = 0.293). Early mAFP implantation was associated with lower mortality in the fully adjusted model (OR, 0.25 [95% CI, 0.06–0.99]; *p* = 0.048). Propensity score-based estimates were directionally consistent, with significance in the doubly robust weighted model (OR, 0.22 [95% CI, 0.06–0.85]; *p* = 0.028) but not in all weighted models. Later-timing and NSTEMI estimates did not reach statistical significance and were imprecise. (4) Conclusions: Among patients with STEMI who survived the first 24 h after ICU admission, mAFP implantation within 6 h was associated with lower subsequent in-hospital mortality. Exploratory later-timing and NSTEMI estimates were inconclusive. These observational findings are hypothesis-generating and require prospective validation.

## 1. Introduction

Acute myocardial infarction complicated by cardiogenic shock (AMI-CS) remains associated with high mortality despite advances in reperfusion therapy, vasoactive support, and intensive care management [1,2,3]. Patients with persistent hypoperfusion, escalating catecholamine requirements, and evolving multiorgan dysfunction are particularly vulnerable. In this setting, temporary mechanical circulatory support (tMCS) is increasingly used to stabilize systemic perfusion and reduce left ventricular workload, but optimal patient selection and timing of support remain incompletely defined [4,5,6,7].

Among available tMCS strategies, microaxial flow pump (mAFP) support provides direct left ventricular unloading while maintaining forward systemic flow. The randomized DanGer Shock trial showed that routine mAFP use in selected patients with ST-segment elevation myocardial infarction (STEMI)-related cardiogenic shock reduced 180-day mortality compared with standard care [8]. Subsequent analyses suggested favorable effects on cardiac loading conditions and pulmonary pressures, supporting the biological plausibility of active ventricular unloading in this setting.

However, the benefit of mAFP support may depend on timing and shock phenotype. Post hoc analyses of DanGer Shock showed that escalation to more advanced tMCS remained necessary in a relevant proportion of patients and was associated with poor outcomes, particularly when escalation occurred after initial standard care [9]. In addition, delay from symptom onset to randomization has been suggested to influence the treatment effect of mAFP support [10]. These observations raise the possibility that mechanical unloading is not only device-dependent, but also time-sensitive.

This question is particularly relevant in routine clinical practice, where AMI-CS is heterogeneous and treatment decisions are often made outside randomized trial conditions. STEMI and non-ST-segment elevation myocardial infarction (NSTEMI) differ in coronary anatomy, ischemic burden, symptom-to-treatment delay, and shock evolution, all of which may modify the association between unloading and outcome [11,12,13]. Whether mAFP implantation timing after intensive care unit (ICU) admission is associated with survival in real-world AMI-CS cohorts remains uncertain.

We therefore investigated the association between mAFP implantation timing and in-hospital mortality in a retrospective single-center cohort of patients with AMI-CS. Using a 24 h landmark design to reduce immortal-time bias, we compared patients receiving mAFP within 6 h after ICU admission with patients managed without tMCS and performed parallel analyses for implantation after 6 h but within 24 h. Because we hypothesized that any survival signal from early unloading would be most apparent in STEMI-related shock, analyses were stratified by STEMI and NSTEMI.

## 2. Materials and Methods

### 2.1. Study Design and Patient Population

This retrospective, single-center observational cohort study was conducted at the Department of Cardiology, Angiology, and Intensive Care Medicine, University Hospital Marburg, Germany. Consecutive adult patients admitted to ICU with AMI-CS between January 2017 and July 2025 were screened for eligibility. The study was approved by the local ethics committee of Philipps University Marburg (approval no. 26-284 RS; 14 August 2026) and was conducted in accordance with the Declaration of Helsinki. Owing to the retrospective design and the use of routinely collected clinical data, the requirement for informed consent was waived.

Cardiogenic shock (CS) was defined at ICU admission based on persistent hypotension with clinical signs of end-organ hypoperfusion requiring vasoactive support and/or tMCS, in line with contemporary ESC-oriented diagnostic frameworks for AMI-related CS [14]. AMI presentation was classified as STEMI or NSTEMI according to contemporary guidelines for acute coronary syndromes [15,16]. The source cohort included patients corresponding clinically to Society for Cardiovascular Angiography and Interventions (SCAI) stage C or higher [17]. For the present analysis, only patients managed either without tMCS or with isolated mAFP support using the Impella CP system (Abiomed, Danvers, MA, USA) were eligible. The study size was determined by the number of consecutive patients meeting the prespecified eligibility criteria during the study period; no formal sample-size calculation was performed. To reduce immortal-time bias, timing analyses were based on a prespecified 24 h landmark after ICU admission. Eligibility for the landmark cohort required survival through the first 24 h; patients who died during this period were excluded. Consequently, all outcome models estimated the association between mAFP timing and subsequent in-hospital mortality from the 24 h landmark onward and do not address mortality during the first 24 h after ICU admission. Patients with mAFP implantation later than 24 h after ICU admission were also excluded from the timing analyses. Within the resulting cohort, mAFP timing was categorized as implantation within 6 h or implantation after 6 h but within 24 h after ICU admission. Patients managed without tMCS served as the reference group for the respective timing comparisons.

### 2.2. Data Collection and Measurements

Clinical, laboratory, procedural, and outcome data were retrospectively extracted from the institutional electronic medical records. ICU admission and mAFP implantation times were obtained from ICU and procedural documentation. Baseline laboratory and hemodynamic variables were defined as values recorded at ICU admission. Left ventricular ejection fraction (LVEF) was obtained from the earliest echocardiographic examination available at ICU admission, and glomerular filtration rate (GFR) was obtained from the corresponding laboratory report. Vasoactive-inotropic score (VIS) was calculated from the vasopressor and inotropic doses documented at ICU admission. Subsequent in-hospital mortality was determined from hospital discharge status. The same data sources and definitions were applied to all treatment groups. Neurological outcome was assessed using the Cerebral Performance Category (CPC) at hospital discharge, with CPC 1–2 defined as a favorable neurological outcome. Patients without a documented CPC assessment were considered unevaluable. Patients were followed from ICU admission until hospital discharge or in-hospital death; no patients were lost to follow-up.

### 2.3. Study Groups, Endpoint, and Covariates

The exposure of interest was mAFP implantation timing after ICU admission. Early implantation was defined as implantation within 6 h after ICU admission, whereas delayed implantation was defined as implantation after 6 h but within 24 h after ICU admission. The 6 h cutoff was prespecified as a pragmatic early-treatment window after ICU admission rather than as a validated biological threshold. It was chosen to distinguish mAFP implantation during the initial phase of shock management from implantation occurring after a more prolonged period of hemodynamic deterioration, while retaining sufficient subgroup sizes for adjusted analyses. Accordingly, the 6 h boundary should be interpreted as an operational definition of early support rather than as evidence of a discrete biological treatment threshold. mAFP implantation was performed at the discretion of the treating interventional cardiology and intensive care team in patients with AMI-CS and evidence of ongoing hypoperfusion or hemodynamic deterioration, including lactate > 2.0 mmol/L, increasing vasopressor or inotropic requirements, persistent hypotension, severely reduced LVEF, or signs of evolving end-organ dysfunction. During the study period, no mandatory institutional protocol specified implantation within or beyond 6 h, and the distinction between early and delayed implantation was applied retrospectively for the present analysis rather than representing a predefined clinical treatment pathway. The decision to initiate mAFP support and its timing were individualized according to the clinical course and therefore subject to operator and team-level variability. Accordingly, four principal subgroup comparisons were evaluated: STEMI with mAFP implantation within 6 h versus no-tMCS, STEMI with implantation after 6 h but within 24 h versus no-tMCS, NSTEMI with implantation within 6 h versus no-tMCS, and NSTEMI with implantation after 6 h but within 24 h versus no-tMCS. The NSTEMI timing analyses were prespecified as exploratory to assess whether a timing-dependent association observed in STEMI might also be present in a clinically distinct AMI-CS phenotype. ICU admission was used as a pragmatic and consistently documented temporal reference point across the cohort; however, in NSTEMI, this time point may occur at variable intervals from ischemic onset and from the onset or progression of CS. Accordingly, the NSTEMI timing categories should be interpreted as operational rather than biological treatment windows. The primary endpoint for all landmark analyses was subsequent in-hospital mortality after the 24 h landmark.

The primary adjustment set was prespecified and consisted of age, sex, prior cardiopulmonary resuscitation (CPR), LVEF at ICU admission, lactate at ICU admission, VIS at ICU admission, GFR at ICU admission, and mean arterial pressure (MAP) at ICU admission. All covariates used for adjustment represented baseline values recorded at ICU admission; no follow-up measurements were included in the adjustment models. These variables were selected to capture demographic risk, cardiac arrest status, left ventricular dysfunction, metabolic shock severity, vasoactive burden, renal function, and systemic perfusion pressure. The same covariates were used both for the main adjusted outcome models and for the estimation of propensity scores in the weighted analyses. Complete-case indicators were generated separately for each subgroup model, and only patients with non-missing values for all variables included in the respective model were entered into adjusted and propensity score-based analyses. The prespecified primary analysis was the STEMI-specific comparison of mAFP implantation within 6 h versus no-tMCS within the 24 h landmark cohort.

### 2.4. Statistical Analysis

Statistical analyses were performed using IBM SPSS Statistics for Windows, version 29.0.2.0 (IBM Corp., Armonk, NY, USA). Continuous variables are presented as mean ± standard deviation or median with interquartile range, as appropriate, and categorical variables as counts and percentages. Crude between-group comparisons of in-hospital mortality were assessed by cross-tabulation using chi-square or Fisher’s exact testing, as appropriate. Multivariable logistic regression models were then fitted in the respective complete-case cohorts to estimate the association between mAFP timing and subsequent in-hospital mortality after the 24 h landmark, with adjustment for the prespecified covariates. Model calibration was assessed using the Hosmer–Lemeshow goodness-of-fit test. A two-sided *p*-value < 0.05 was considered statistically significant. To further address baseline differences between treatment groups, propensity score-based weighted analyses were performed separately for each timing comparison. Propensity scores were estimated using binary logistic regression with treatment assignment as the dependent variable and the same prespecified baseline covariates as predictors: age, sex, prior CPR, LVEF, lactate, VIS, GFR, and MAP at ICU admission. Stabilized inverse probability of treatment weights (IPTWs) were calculated using the marginal probability of the observed treatment divided by the individual propensity score in treated patients and the marginal probability of no treatment divided by one minus the propensity score in untreated patients. Extreme weights were trimmed at the 1st and 99th percentiles. Covariate balance before and after weighting was assessed using absolute standardized mean differences (SMDs). An absolute SMD < 0.10 was considered indicative of acceptable covariate balance. Balance diagnostics for the primary STEMI mAFP ≤ 6 h versus no-tMCS comparison were visualized in a Love plot using the final stabilized and trimmed IPTWs. Weighted analyses included a treatment-only IPTW model to estimate the weighted marginal treatment association and a doubly robust weighted logistic regression model additionally adjusted for the same prespecified covariates. As a sensitivity analysis, overlap weighting was also performed, assigning treated patients a weight of one minus the propensity score and untreated patients a weight equal to the propensity score. Overlap-weighted treatment-only and covariate-adjusted models were estimated analogously.

## 3. Results

### 3.1. Study Population

Between January 2017 and July 2025, 479 patients with AMI-CS were screened. Of these, 123 patients were excluded from the prespecified comparison framework because they were treated with venoarterial extracorporeal membrane oxygenation (VA-ECMO) (*n* = 59) or with combined support (ECMELLA) (*n* = 64), leaving 356 patients managed either without tMCS or with isolated mAFP support. After application of the 24 h landmark and timing criteria, 74 additional patients were excluded because of death within the first 24 h (*n* = 38) or mAFP implantation later than 24 h after ICU admission (*n* = 36). Among the 38 patients who died within the first 24 h, 18 had been managed without tMCS and 20 had received isolated mAFP support; no fatal mAFP-related complication was identified among these patients. The final timing cohort comprised 282 patients and was stratified by infarct type and mAFP timing as shown in Figure 1.

### 3.2. Baseline Characteristics of the Primary STEMI Cohort

Baseline clinical and angiographic characteristics of STEMI patients receiving mAFP within 6 h after ICU admission and those managed without tMCS are shown in Table 1. Patients treated with early mAFP were younger and less frequently had a history of stroke, whereas sex distribution, body mass index, overall comorbidity burden, and most cardiovascular risk factors were broadly similar between groups. Coronary disease extent did not differ significantly, and final post-PCI TIMI 3 flow was comparable. However, multivessel PCI was performed more frequently in the early mAFP group.

Resuscitation characteristics, organ support, ICU admission physiology, and established risk scores are summarized in Table 2. Rates of CPR, shockable rhythm, invasive mechanical ventilation, and continuous renal replacement therapy (CRRT) were not significantly different between groups. ICU admission lactate, pH, VIS, Sequential Organ Failure Assessment (SOFA) score, Horovitz index, and CardShock score were also comparable, suggesting a broadly similar early shock phenotype by conventional severity markers. Despite comparable markers of systemic hypoperfusion and shock severity, patients receiving early mAFP had significantly lower LVEF, indicating more pronounced left ventricular dysfunction. Conversely, renal function and hemoglobin levels were more preserved in the early mAFP group. Overall, baseline characteristics suggested a mixed clinical profile rather than uniform low-risk selection for early mAFP, supporting the use of adjusted and propensity score-based analyses for outcome assessment.

To further explore potential treatment-selection patterns, STEMI patients receiving mAFP within 6 h were directly compared with those receiving mAFP after 6 h but within 24 h (Supplementary Table S1). Baseline demographic, comorbidity, angiographic, organ-support, ICU admission physiology, and established shock-severity characteristics did not differ significantly between groups. The only nominally significant difference was a higher frequency of shockable initial rhythm in the early-treatment group (29.8% vs. 0%; *p* = 0.034). Given the small delayed-treatment subgroup (*n* = 11), these comparisons were considered exploratory and should be interpreted cautiously.

### 3.3. Clinical Outcomes and Treatment-Course Variables

Clinical outcomes and treatment-course variables are summarized in Table 3. Unadjusted in-hospital mortality was numerically lower in patients treated with early mAFP than in patients managed without tMCS, although this difference was not statistically significant. Favorable neurological outcome among evaluable patients differed in the unadjusted comparison, but this finding was interpreted cautiously because neurological outcome was not the primary endpoint and was available only in evaluable patients. Duration of mechanical ventilation and duration of CRRT did not differ significantly between groups. Hospital length of stay was longer in the early mAFP group; however, length-of-stay and organ-support duration variables were considered descriptive because they are influenced by survival and the post-baseline treatment course.

### 3.4. Adjusted Association Between mAFP Timing and In-Hospital Mortality

In the primary STEMI-specific 24 h landmark cohort, which by design comprised patients alive 24 h after ICU admission, LVEF was missing in 8 patients, lactate in 12, GFR in 2, and MAP in 5, whereas age, sex, prior CPR, and VIS were complete. Because missingness overlapped across variables, complete-case analysis was available in 139 of 165 patients (84.2%). In the fully adjusted multivariable logistic regression model, mAFP implantation within 6 h after ICU admission was associated with lower subsequent in-hospital mortality from the landmark onward (OR 0.25, 95% CI 0.06–0.99; *p* = 0.048). To further account for baseline differences, propensity scores were estimated using the same prespecified covariates as in the adjusted model and were applied in stabilized IPTW and overlap-weighted sensitivity analyses. In the stabilized IPTW analysis, the treatment-only weighted model showed a directionally consistent but non-significant association (OR 0.45, 95% CI 0.19–1.07; *p* = 0.071), whereas the doubly robust weighted model yielded a statistically significant estimate in the same direction (OR 0.22, 95% CI 0.06–0.85; *p* = 0.028). Overlap-weighted models were directionally consistent but did not reach statistical significance. Stabilized and trimmed IPTW improved covariate balance for most prespecified variables. Absolute SMDs after weighting were below 0.10 for sex, prior CPR, LVEF, lactate, GFR, and MAP, whereas residual imbalance remained for age and VIS (Supplementary Figure S1). In the exploratory later-timing and NSTEMI cohorts, none of the adjusted associations reached statistical significance. In STEMI patients receiving mAFP after 6 h but within 24 h after ICU admission, crude subsequent in-hospital mortality was 18.2% compared with 23.7% in the corresponding no-tMCS group (*p* = 0.688), and the fully adjusted model showed no statistically significant association (OR 1.73, 95% CI 0.15–20.03; *p* = 0.663). In NSTEMI patients receiving mAFP within 6 h after ICU admission, crude subsequent in-hospital mortality was 31.3% compared with 31.9% in the no-tMCS group (*p* = 0.950), and the adjusted estimate did not reach statistical significance (OR 8.03, 95% CI 0.70–92.18; *p* = 0.094). Similarly, in NSTEMI patients receiving mAFP after 6 h but within 24 h after ICU admission, crude subsequent in-hospital mortality was 37.0% compared with 31.9% in the no-tMCS group (*p* = 0.654), and the adjusted estimate did not reach statistical significance in the corresponding complete-case cohort (OR 3.18, 95% CI 0.60–16.83; *p* = 0.175). Propensity score-based sensitivity analyses in these exploratory cohorts likewise did not yield statistically significant estimates. The adjusted subgroup estimates are summarized in Figure 2.

### 3.5. Out-of-Hospital CPR Subgroup Analysis

In exploratory analyses restricted to patients with out-of-hospital CPR within the primary STEMI 24 h landmark cohort, early mAFP implantation was associated with numerically lower crude in-hospital mortality compared with no-tMCS (12.0% vs. 25.0%; *p* = 0.257). However, no significant between-group differences were observed in early hemodynamic or metabolic recovery, as reflected by 24 h VIS (B = −6.037; *p* = 0.302) and 24 h lactate (B = 0.915; *p* = 0.111). Given the small subgroup size and instability of adjusted mortality modelling, these findings should be interpreted as exploratory and hypothesis-generating only.

## 4. Discussion

In this real-world cohort of patients with AMI-CS, mAFP implantation within 6 h after ICU admission was associated with lower subsequent in-hospital mortality among STEMI patients who survived the first 24 h. The direction of the estimate was broadly consistent across multivariable and propensity score-based analyses, although not all weighted models reached statistical significance. In the exploratory later-timing and NSTEMI analyses, estimates were imprecise and non-significant and should not be interpreted as demonstrating the absence of an association. Taken together, these observational findings provide a hypothesis-generating signal that the association between mAFP support and subsequent mortality may depend on timing and infarct presentation.

Our findings are consistent with the central concept of the DanGer Shock trial, which demonstrated improved survival with mAFP support in carefully selected patients with STEMI-related CS [8]. However, comparison with DanGer Shock should be made cautiously because that trial assessed 180-day mortality, whereas the present analysis was restricted to in-hospital mortality and therefore captures an earlier outcome period. Subsequent analyses from DanGer Shock further support the biological plausibility of early unloading by showing favorable effects on cardiac loading conditions and by highlighting the importance of patient selection and shock trajectory [18,19]. In the DanGer Shock escalation analysis, escalation to higher-flow tMCS occurred in nearly one-fifth of patients, was more frequent in the control group, and was associated with markedly higher mortality, particularly when escalation occurred after initial standard care [9,20]. This supports the concept that late rescue strategies may identify patients already on a deteriorating trajectory rather than patients in whom unloading can still modify outcome.

The absence of a signal in the >6–≤24 h STEMI cohort is therefore clinically relevant. It argues against a simple “device-versus-no-device” interpretation and suggests that timing may be central to the treatment effect [21,22]. Once progressive hypoperfusion, catecholamine escalation, acidosis, and organ dysfunction are established, later unloading may no longer be sufficient to change the clinical course [23,24]. In this regard, our results support a time-sensitive treatment concept rather than indiscriminate mAFP implantation during the first 24 h.

This interpretation is important when comparing our findings with the recent national analysis by Desai et al. In that study, mAFP placement within 24 h in AMI-CS was associated with higher adjusted odds of in-hospital mortality, invasive mechanical ventilation, major bleeding, and dialysis-requiring acute kidney injury [25]. These results appear to contrast with our primary STEMI finding. However, the studies differ substantially. The national analysis used an administrative 24 h timing window, whereas our signal was restricted to implantation within 6 h and was absent in the later >6–≤24 h window. In addition, our cohort used granular clinical variables, excluded VA-ECMO and ECMELLA to create an isolated mAFP versus no-tMCS comparison, and applied a 24 h landmark design. Thus, the two studies may not be contradictory, but rather complementary: broad mAFP use within an administrative 24 h window may reflect confounding by severity and non-selective use, whereas early implantation in a selected STEMI subgroup with marked left ventricular dysfunction but without more advanced systemic shock markers may identify patients more likely to benefit.

The phenotype-based DanGer Shock analysis also supports this interpretation. In that study, mortality and treatment response differed substantially across CS phenotypes, with the greatest apparent benefit of mAFP observed in the non-congested phenotype and no clear benefit in cardiorenal shock [26]. Although we did not formally assign shock phenotypes, the primary STEMI cohort showed a mixed clinical–hemodynamic profile. Patients receiving early mAFP had more pronounced LV systolic dysfunction, whereas lactate, pH, VIS, SOFA score, Horovitz index, and CardShock score were broadly comparable, and the GRACE score was lower. This apparent divergence is not necessarily contradictory because LVEF reflects LV systolic dysfunction, whereas the GRACE score integrates age, hemodynamic status, renal function, cardiac arrest, electrocardiographic changes, and cardiac biomarkers without incorporating LVEF. The younger age and more preserved renal function of early-mAFP patients likely contributed to their lower GRACE scores despite poorer LV function. This pattern may reflect selective use of mAFP in patients with severe LV dysfunction but greater systemic reserve; however, it also reinforces the possibility of residual confounding by treatment selection.

The lack of a significant association in NSTEMI should be interpreted cautiously. NSTEMI-related CS is clinically heterogeneous, with less clearly defined ischemic timing, more frequent multivessel disease, and more variable mechanisms of deterioration [27,28]. In contrast to STEMI, ICU admission in NSTEMI may occur at highly variable intervals from ischemic onset and from the onset or progression of CS. Thus, the ≤6 h and >6–≤24 h categories reflect time from a pragmatic clinical reference point rather than a biologically uniform treatment window. This temporal heterogeneity may attenuate or obscure timing-dependent associations. Accordingly, the NSTEMI analyses should be regarded as exploratory and do not exclude a potential role for mAFP in selected NSTEMI-CS patients.

Finally, the recent DanGer Shock fast-recovery analysis highlights the challenge of patient selection. Approximately one-third of patients in the standard-care arm recovered rapidly, had low mortality, and were characterized by younger age, higher LVEF, and lower lactate, although prediction at the individual level remained modest [29]. This is clinically important because routine mAFP use may expose some patients to unnecessary device-related risk, whereas delayed support may miss the therapeutic window in others. Our data support this nuanced view: early mAFP was associated with lower adjusted mortality in selected STEMI patients, but this signal was not universal across timing windows or infarct types.

Overall, our findings are consistent with the hypothesis that the association between mAFP support and outcome may depend on patient selection and treatment timing. Rather than asking whether mechanical unloading should be used broadly, future studies should define which patients benefit, how early unloading must be initiated, and which clinical or hemodynamic phenotypes require alternative or escalated support strategies.

## 5. Limitations

This study has several limitations. The retrospective, single-center design limits causal inference and generalizability, and treatment allocation was not randomized but based on clinical decision-making in routine care. Although multivariable adjustment, propensity score-based models, and a 24 h landmark design were used to reduce confounding and immortal-time bias, residual confounding and treatment-selection bias cannot be excluded. In particular, patients receiving early mAFP were younger and had more preserved renal function, whereas VIS also differed numerically between groups; these factors may independently influence mortality. Moreover, residual imbalance in age and VIS persisted after stabilized IPTW. Conversely, LVEF was lower in the early-mAFP group, indicating that baseline differences were not uniformly favorable. Accordingly, the observed association should not be interpreted as establishing an independent causal effect of early mAFP implantation. The landmark approach also restricts interpretation to patients who survived the first 24 h after ICU admission and therefore does not capture the sickest patients who died very early. Consequently, the reported estimates concern subsequent in-hospital mortality from the landmark onward rather than mortality in the full AMI-CS population from admission. Timing was defined from ICU admission rather than from symptom onset, shock onset, or revascularization, which may be particularly relevant in NSTEMI patients with less clearly defined ischemic timing. Furthermore, the 6 h cutoff was an operationally defined threshold and should not be interpreted as evidence of a discrete biological treatment window. The delayed STEMI and NSTEMI timing cohorts were relatively small, resulting in wide confidence intervals and limited statistical power. Their non-significant estimates should therefore not be interpreted as evidence of no association, and these subgroup analyses should be considered exploratory and hypothesis-generating. In addition, missing data required complete-case analyses. Indications and timing of mAFP implantation were individualized and not governed by a mandatory institutional protocol, resulting in potential operator-dependent treatment selection. The precise temporal relationship of mAFP implantation to PCI was not systematically available for reliable classification, and device management, escalation strategies, and weaning were not standardized. Systematic invasive hemodynamic data and formal shock phenotype classification were not available, limiting mechanistic interpretation. Finally, by design, patients treated with VA-ECMO or ECMELLA were excluded to allow a clinically coherent comparison of isolated mAFP support with no-tMCS. This exclusion removed a substantial subgroup likely to include patients with more advanced shock and limits generalizability to the full severity spectrum of AMI-CS encountered in clinical practice. Accordingly, our findings should not be extrapolated to patients requiring extracorporeal or combined tMCS.

## 6. Conclusions

Among patients with STEMI-related cardiogenic shock who survived the first 24 h after ICU admission, mAFP implantation within 6 h was associated with lower subsequent in-hospital mortality. Later-timing and NSTEMI estimates were imprecise and inconclusive. These observational findings are hypothesis-generating and require prospective validation.

## Figures and Tables

**Figure 1:**
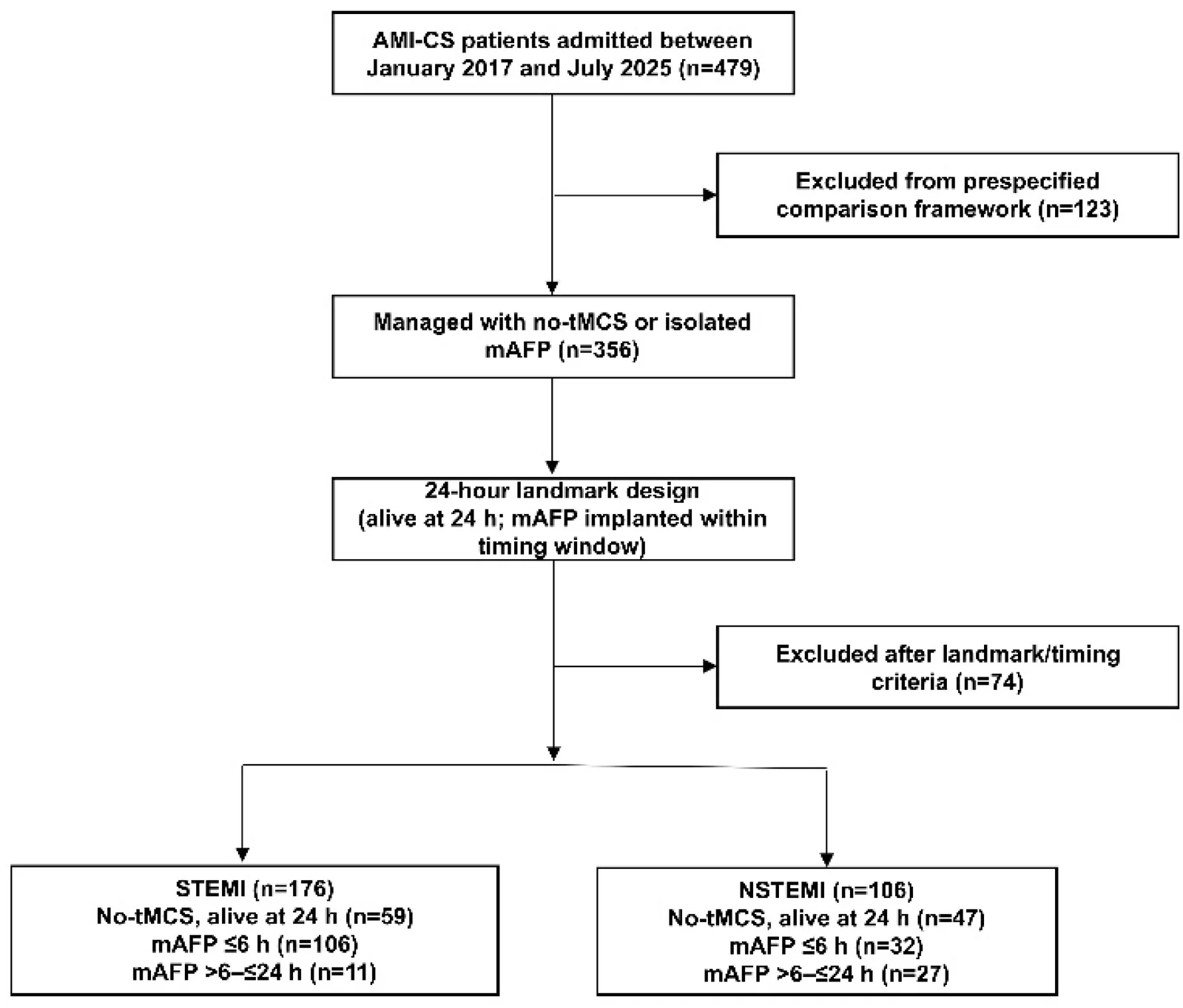
Study flowchart. Abbreviations: AMI-CS, acute myocardial infarction-related cardiogenic shock; mAFP, microaxial flow pump; NSTEMI, non-ST-segment elevation myocardial infarction; STEMI, ST-segment elevation myocardial infarction; tMCS, temporary mechanical circulatory support.

**Figure 2:**
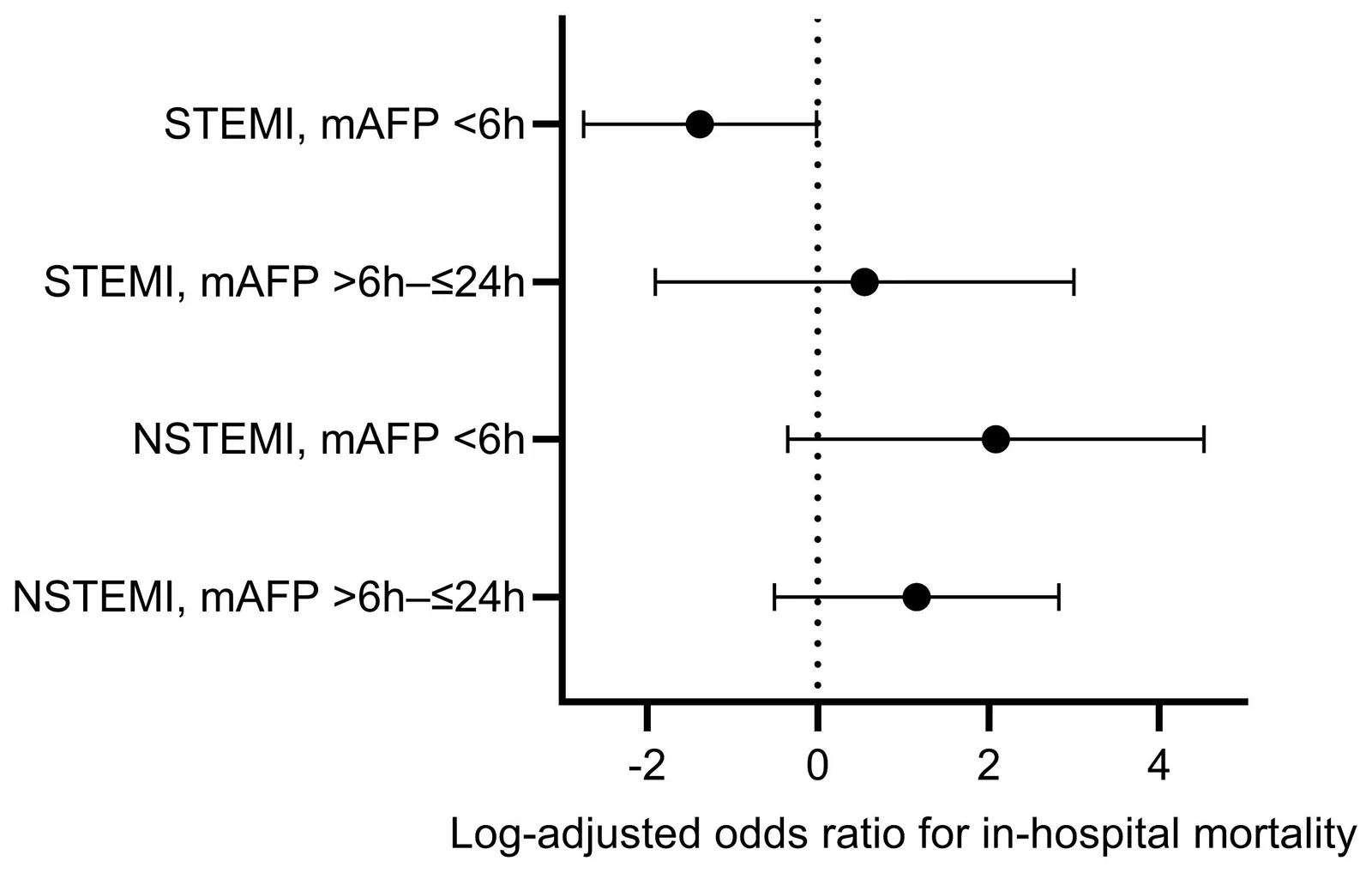
Forest plot of fully adjusted odds ratios for in-hospital mortality across prespecified STEMI and NSTEMI timing cohorts. Odds ratios compare each mAFP timing group with the corresponding no-tMCS group within the 24 h landmark cohort. Estimates are displayed on the log(OR) scale, where 0 corresponds to an OR of 1.0; values below 0 indicate lower odds of in-hospital mortality, and values above 0 indicate higher odds of in-hospital mortality. Abbreviations: mAFP, microaxial flow pump; NSTEMI, non-ST-segment elevation myocardial infarction; OR, odds ratio; STEMI, ST-segment elevation myocardial infarction.

**Table 1:** Baseline clinical, comorbidity, and angiographic characteristics of STEMI patients treated with mAFP within 6 h after ICU admission versus STEMI patients managed without tMCS in the 24 h landmark cohort.

Variable	STEMI, mAFP ≤ 6 h (*n* = 106)	No-tMCS (*n* = 59)	*p*-Value
Demographics and comorbidities			
Age (years) ^2^	66.07 (±11.39)	71.64 (±11.79)	0.003
Male sex ^1^	84 (79.2)	40 (67.8)	0.103
BMI ^2^	28.43 (±4.79)	26.96 (±4.27)	0.064
Charlson comorbidity index ^2^	3.31 (±2.21)	3.98 (±2.25)	0.064
Known coronary artery disease ^1^	21 (19.8)	9 (15.3)	0.467
Prior myocardial infarction ^1^	16 (15.1)	9 (15.3)	0.978
Atrial fibrillation ^1^	7 (6.6)	8 (13.6)	0.136
Arterial hypertension ^1^	55 (51.9)	38 (64.4)	0.120
Diabetes mellitus ^1^	35 (33.0)	13 (22.0)	0.136
Chronic kidney disease (KDIGO stage ≥ 3a) ^1^	10 (9.4)	8 (13.6)	0.415
Prior stroke ^1^	3 (2.8)	8 (13.6)	0.008
Peripheral artery disease ^1^	7 (6.7)	6 (10.2)	0.426
Coronary angiography and infarct characteristics			
Single-vessel coronary artery disease ^1^	28 (26.4)	17 (28.8)	0.740
Multivessel coronary artery disease ^1^	78 (73.6)	42 (71.2)	0.740
Single-vessel PCI ^1^	80 (75.5)	53 (89.8)	0.025
Multivessel PCI ^1^	26 (24.5)	6 (10.2)	0.025
Post-PCI TIMI 3 flow ^1^	102 (96.2)	51 (94.4)	0.602

Data are shown as ^1^ n (%), or ^2^ mean ± standard deviation, as appropriate. Abbreviations: mAFP, microaxial flow pump; tMCS, temporary mechanical circulatory support; BMI, body mass index; PCI, percutaneous coronary intervention; KDIGO, Kidney Disease: Improving Global Outcomes; TIMI, Thrombolysis in Myocardial Infarction.

**Table 2:** Resuscitation characteristics, organ support, ICU admission physiology, and severity scores of STEMI patients treated with mAFP within 6 h after ICU admission versus STEMI patients managed without tMCS in the 24 h landmark cohort.

Variable	STEMI, mAFP ≤ 6 h (*n* = 106)	No-tMCS (*n* = 59)	*p*-Value
Resuscitation characteristics			
CPR ^1^	42 (39.6)	28 (47.5)	0.329
Out-of-hospital CPR ^1^	25 (23.6)	20 (33.9)	0.154
Shockable rhythm at initial rhythm assessment (VF/pVT) ^1^	31 (29.8)	20 (33.9)	0.588
Organ support therapies			
Invasive mechanical ventilation ^1^	56 (52.8)	34 (57.6)	0.553
CRRT ^1^	31 (29.2)	11 (18.6)	0.134
Parameters at ICU admission			
Left ventricular ejection fraction (%) ^2^	36.13 (±8.76)	45.16 (±10.92)	<0.001
Lactate (mmol/L) ^3^	2.45 (1.38–4.30)	2.80 (1.70–5.10)	0.220
pH ^3^	7.34 (7.29–7.39)	7.36 (7.32–7.41)	0.213
Creatinine (mg/dL) ^3^	1.08 (0.90–1.33)	1.21 (0.99–1.40)	0.041
Estimated glomerular filtration rate (mL/min/1.73 m^2^) ^3^	64.0 (51.8–82.2)	57.0 (41.0–67.5)	0.009
Hemoglobin (g/L) ^3^	145.0 (127.0–157.0)	133.0 (123.0–142.5)	<0.001
Leukocyte count (/nL) ^3^	13.80 (11.00–17.55)	14.00 (10.70–17.00)	0.612
Platelet count (/nL) ^3^	238.5 (197.5–301.5)	255.5 (197.5–322.0)	0.496
C-reactive protein (mg/L) ^3^	4.55 (2.10–11.35)	7.40 (2.70–21.25)	0.147
Procalcitonin (ng/mL) ^3^	0.10 (0.10–0.10)	0.10 (0.10–0.20)	0.416
VIS ^3^	6.60 (3.57–21.34)	12.14 (4.83–20.36)	0.284
Risk and severity scores at ICU admission			
SOFA score ^2^	6.42 (±4.02)	6.53 (±3.40)	0.851
Horovitz index (mmHg) ^3^	330.3 (163.8–481.0)	386.0 (198.0–713.1)	0.088
CardShock score ^2^	2.94 (±1.76)	2.96 (±1.59)	0.937
IABP-SHOCK II score ^2^	1.58 (±1.49)	2.12 (±1.86)	0.057
GRACE score ^2^	177.70 (±26.43)	189.15 (±25.01)	0.015

Data are shown as ^1^ n (%), ^2^ mean ± standard deviation, or ^3^ median (interquartile range), as appropriate. Abbreviations: mAFP, microaxial flow pump; tMCS, temporary mechanical circulatory support; CPR, cardiopulmonary resuscitation; VF, ventricular fibrillation; pVT, pulseless ventricular tachycardia; CRRT, continuous renal replacement therapy; ICU, intensive care unit; VIS, vasoactive-inotropic score; SOFA, Sequential Organ Failure Assessment; IABP, intra-aortic balloon pump; GRACE, Global Registry of Acute Coronary Events.

**Table 3:** Clinical outcomes and treatment-course variables in STEMI patients treated with mAFP within 6 h after ICU admission versus STEMI patients managed without tMCS in the 24 h landmark cohort.

Variable	STEMI, mAFP ≤ 6 h (*n* = 106)	No-tMCS (*n* = 59)	*p*-Value
Clinical outcomes and treatment-course variables			
In-hospital mortality ^1^	18 (17.0)	14 (23.7)	0.293
Favorable neurological outcome (CPC 1–2), among evaluable patients ^1^	60 (68.2)	37 (86.0)	0.029
Duration of mechanical ventilation (h) ^3^	57.5 (0.0–277.5)	61.0 (0.0–169.0)	0.503
Duration of CRRT (h) ^3^	0.0 (0.0–54.0)	0.0 (0.0–0.0)	0.081
Hospital length of stay (days) ^2^	15.79 (±11.30)	12.08 (±6.40)	0.008

Data are shown as ^1^ n (%), ^2^ mean ± standard deviation, or ^3^ median (interquartile range), as appropriate. Length-of-stay and organ-support variables are reported descriptively and were not considered primary endpoints. Abbreviations: CPC, Cerebral Performance Category; CRRT, continuous renal replacement therapy; ICU, intensive care unit; mAFP, microaxial flow pump; STEMI, ST-segment elevation myocardial infarction; tMCS, temporary mechanical circulatory support.

## Data Availability

The data that support the findings of this study are available from the corresponding author upon reasonable request, subject to applicable ethical and data-protection restrictions.

## Supplementary Materials

The following supporting information can be downloaded at: https://www.mdpi.com/article/10.3390/jcdd13100498/s1, Figure S1: Covariate balance before and after stabilized inverse probability of treatment weighting in the primary STEMI mAFP ≤6-h versus no-tMCS comparison; Table S1: Direct comparison of clinical characteristics between STEMI patients receiving isolated mAFP within 6 h versus >6–≤24 h after ICU admission in the 24-h landmark cohort.
