# Supplementary material for: Timing of Microaxial Flow Pump Support and In-Hospital Mortality in Infarct-Related Cardiogenic Shock: The TIMING-CS Study
Source: J Cardiovasc Dev Dis. 2026 Oct 3;13(10):498. doi: 10.3390/jcdd13100498 (PMC13705220; doi:10.3390/jcdd13100498)
Supplement: Supplementary file 1 [file jcdd-13-00498-s001.zip › jcdd-4554669-supplementary.pdf]

Supplement

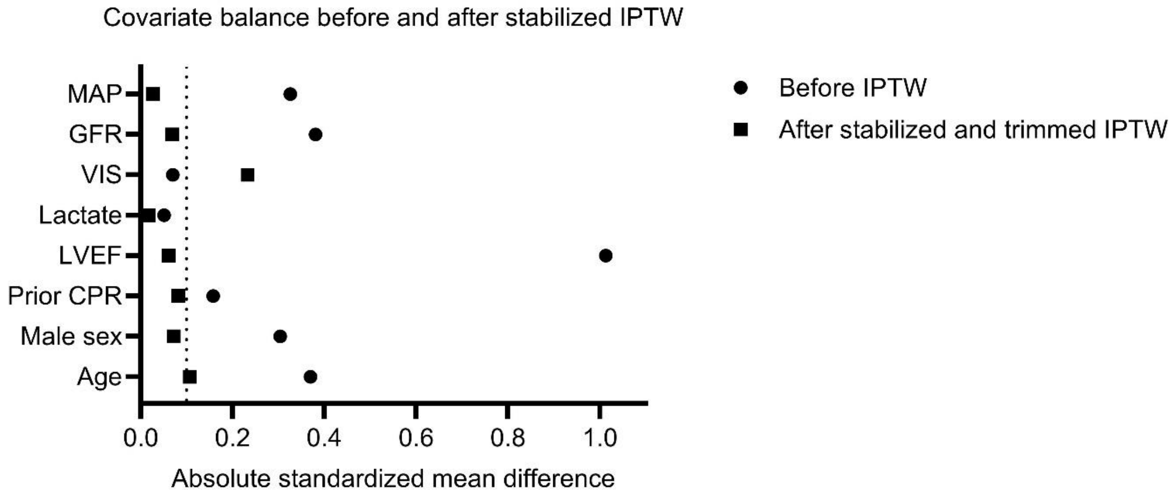

Supplementary Figure S1. Covariate balance before and after stabilized inverse probability of treatment weighting in the primary STEMI mAFP ≤6-hour versus no-tMCS comparison. Absolute standardized mean differences are shown before weighting and after application of the final stabilized and trimmed IPTW weights. The vertical dotted line at 0.10 indicates the threshold for acceptable covariate balance. Abbreviations: CPR, cardiopulmonary resuscitation; GFR, glomerular filtration rate; IPTW, inverse probability of treatment weighting; LVEF, left ventricular ejection fraction; MAP, mean arterial pressure; SMD, standardized mean difference; VIS, vasoactive-inotropic score.

Supplementary Table S1. Direct comparison of clinical characteristics between STEMI patients receiving isolated mAFP within 6 hours versus >6–≤24 hours after ICU admission in the 24-hour landmark cohort. Data are shown as [1] n (%), [2] mean ± standard deviation, or [3] median (interquartile range), as appropriate. Abbreviations: BMI, body mass index; CPR, cardiopulmonary resuscitation; CRRT, continuous renal replacement therapy; eGFR, estimated glomerular filtration rate; GRACE, Global Registry of Acute Coronary Events; IABP, intra-aortic balloon pump; KDIGO, Kidney Disease: Improving Global Outcomes; LVEF, left ventricular ejection fraction; mAFP, microaxial flow pump; PCI, percutaneous coronary intervention; pVT, pulseless ventricular tachycardia; SOFA, Sequential Organ Failure Assessment; TIMI, Thrombolysis in Myocardial Infarction; VF, ventricular fibrillation; VIS, vasoactive-inotropic score.

Variable	mAFP ≤6 h (n=106)	mAFP >6–≤24 h (n=11)	p-value
**Demographics and comorbidities**			
Age (years)[2]	66.07 ± 11.39	69.27 ± 11.62	0.377
Male sex[1]	84 (79.2)	7 (63.6)	0.259
BMI[2]	28.43 ± 4.79	27.27 ± 3.58	0.438
Charlson comorbidity index[2]	3.31 ± 2.21	4.18 ± 2.60	0.223
Known coronary artery disease[1]	21 (19.8)	4 (36.4)	0.245
Prior myocardial infarction[1]	16 (15.1)	4 (36.4)	0.093
Atrial fibrillation[1]	7 (6.6)	0 (0.0)	1.000
Arterial hypertension[1]	55 (51.9)	7 (63.6)	0.537
Diabetes mellitus[1]	35 (33.0)	3 (27.3)	1.000
Chronic kidney disease (KDIGO stage ≥3a)[1]	10 (9.4)	1 (9.1)	1.000
Prior stroke[1]	3 (2.8)	1 (9.1)	0.330
Peripheral artery disease[1]	7 (6.7)	1 (9.1)	0.561

**Coronary angiography and infarct characteristics**			
Single-vessel coronary artery disease[1]	28 (26.4)	1 (9.1)	0.288
Multivessel coronary artery disease[1]	78 (73.6)	10 (90.9)	0.288
Single-vessel PCI[1]	80 (75.5)	7 (63.6)	0.469
Multivessel PCI[1]	26 (24.5)	4 (36.4)	0.469
Post-PCI TIMI 3 flow[1]	102 (96.2)	11 (100.0)	1.000
**Resuscitation characteristics and organ support**			
CPR[1]	42 (39.6)	1 (9.1)	0.053
Out-of-hospital CPR[1]	25 (23.6)	0 (0.0)	0.117
Shockable initial rhythm (VF/pVT)[1]	31 (29.8)	0 (0.0)	0.034
Invasive mechanical ventilation[1]	56 (52.8)	4 (36.4)	0.354
CRRT[1]	31 (29.2)	4 (36.4)	0.731
**Parameters at ICU admission**			
LVEF (%)[2]	36.13 ± 8.76	41.18 ± 11.76	0.082
Lactate (mmol/L)[3]	2.45 (1.33–4.30)	1.90 (1.30–2.00)	0.284
pH[3]	7.34 (7.29–7.39)	7.38 (7.29–7.42)	0.295
Creatinine (mg/dL)[3]	1.08 (0.89–1.34)	0.90 (0.74–1.25)	0.435
eGFR (mL/min/1.73 m²)[3]	64.0 (51.3–82.8)	62.0 (41.0–86.0)	0.779
Hemoglobin (g/L)[3]	145.0 (127.0–157.3)	147.0 (127.0–153.0)	0.855
Leukocyte count (/nL)[3]	13.80 (10.98–17.60)	13.60 (11.10–16.70)	0.783
Platelet count (/nL)[3]	238.5 (195.8–302.5)	207.0 (184.0–260.0)	0.156
C-reactive protein (mg/L)[3]	4.55 (2.03–11.98)	11.90 (2.30–30.10)	0.173
VIS[3]	6.60 (3.56–21.76)	3.47 (0.00–7.72)	0.133
**Risk and severity scores at ICU admission**			
SOFA score[2]	6.42 ± 4.02	4.91 ± 4.16	0.240
Horovitz index (mmHg)[3]	330.3 (160.0–481.0)	362.9 (199.5–636.9)	0.554
CardShock score[2]	2.94 ± 1.77	2.45 ± 1.81	0.395
IABP-SHOCK II score[2]	1.58 ± 1.49	1.18 ± 1.33	0.398
GRACE score[2]	177.70 ± 26.43	181.00 ± 28.04	0.698
